# Research on the restorative effects and perception of privacy driven activities in Chinese classical gardens—Case studies of three Suzhou gardens

**DOI:** 10.3389/fpubh.2024.1462077

**Published:** 2024-10-15

**Authors:** Hanxin Liu, Guoshu Bin, Xiao Wang, Jia Luo, Minyi Liu

**Affiliations:** ^1^College of Tourism & Landscape Architecture, Guilin University of Technology, Guilin, China; ^2^Institute of Guangxi Tourism Industry, Guilin, China; ^3^College of Architecture, Soochow University, Suzhou, China

**Keywords:** private activities, classical garden, restorative effect, spatial perception, physiological feedback

## Abstract

The need for privacy, recognized as a fundamental psychological requirement, has garnered increasing attention as researchers explore the restorative effects of privacy driven activities (PDA). This study employs quantitative experiments and analyses to assess the restorative benefits of PDA within three classical Chinese gardens, demonstrating their superiority over conventional leisure activities in promoting emotional recovery, reducing stress, and restoring attention. The experiment quantifies the restorative effects of PDA versus standard leisure activities using a classic restorative scale and physiological indicators reflecting emotional relief. Regression analyses then identify five key factors influencing the occurrence of PDA, derived from preferred locations and behavioral tendencies observed in the three gardens. Further analyses reveal significant differences in the impacts of these five environmental feature dimensions on the evaluation indicators of “preference for privacy-oriented activities” and “restorative effects”. Among these dimensions, “spatial scale and accessibility” has the greatest impact on the “preference for privacy-oriented environments”, while “spatial atmosphere” and “activity facilities” have the most significant impact on “restorative effects”. The findings suggest that behavioral activities mediate the relationship between environmental factors and restorative effects, highlighting the potential of PDA as a mediating variable for a comprehensive investigation into the pathways and mechanisms influencing restorative environments in research and design.

## 1 Introduction

The health values of human settlement environment have been widely recognized and studied. Globally, researchers have established that, compared to the built environment, the natural environment is more effective in reducing stress and enhancing emotional status (1–3), and in promoting physical health (4, 5); this could be a result of the fact that humans possess biophilia, an innate tendency to seek connections with nature, and that engaging with nature provides restorative experiences (6). It has also been discovered that individual emotional reactions and the activation of internal cognitive processes are influenced differently by various settings (7). Among these settings, Chinese classical gardens stand out as remarkable examples of human-made natural environments, or, put another way, “miniature nature”. Being one of the three oldest and most prestigious garden types in the world, Chinese classical gardens are renowned for their nature-inspired design philosophy and represent a distinctive spatial environment that seamlessly combines traditional cultural elements, aesthetic values, and recreational contemplation (8). Therefore, Chinese classical gardens are particularly valuable for studying the “biophilic” mechanisms through which privacy is experienced in natural or nature-like spaces.

From an environmental behavioral perspective, it is understood that the environment can either facilitate or hinder the development of behavior. The interaction between the environment and behavior plays a crucial role in promoting health and healing (9, 10). Research has also shown that various behavioral factors, such as activity type, duration, and frequency, are linked to an individual's physical and mental wellbeing. Moreover, the therapeutic benefits of privacy-related activities have been evident for years. Newell's privacy systematization model illustrates how different privacy features systematically support and enhance individuals, fulfilling their functional value (11). Environmental psychologist Pedersen conducted extensive research, demonstrating the significant restorative function of privacy-related activities (12). Liu's work has revealed that engaging in privacy activities within urban public spaces can enhance spatial vitality and engagement (13, 14). Furthermore, studies have confirmed that different urban landscape settings and sceneries can have varying impacts on the restoration process (15). Notably, a review of the existing literature highlights that research on the restorative effects of environmental behavior has predominantly focused on natural environments and urban landscapes, often overlooking a distinctive built landscape type—Chinese classical gardens.

However, studies have been carried out mainly on qualitative health restorative research in terms of landscape elements and techniques, and there is a lack of specific research on the perception of specific restorative behaviors in the garden environment. Therefore, this study explores the linkage effect between restorative environments and restorative behaviors by introducing PDA into the classical garden environment, providing a more precise way to study restorative environment creation, also provides a new perspective and typical reference value for the creation of “garden-like” high-quality urban spaces in the context of our own spatial and cultural traditions and aesthetic aspirations.

For the above research objectives, this study selected the Master of Nets Garden, the Lingering Garden, and the Lion Grove Garden from Suzhou city to apply a quantitative research method examining spatial environmental characteristics, PDA and restorative effects. This research aims to answer the following questions:

1) How to verify the restorative effects of PDAs in classical gardens?
2) How to identify the key environmental factors influencing restorative effects in classical gardens?
3) Is there a difference in the impact pathways of key environmental factors on the formation of privacy and restorative effects?

## 2 Research design and data analyses

**Figure 1:**
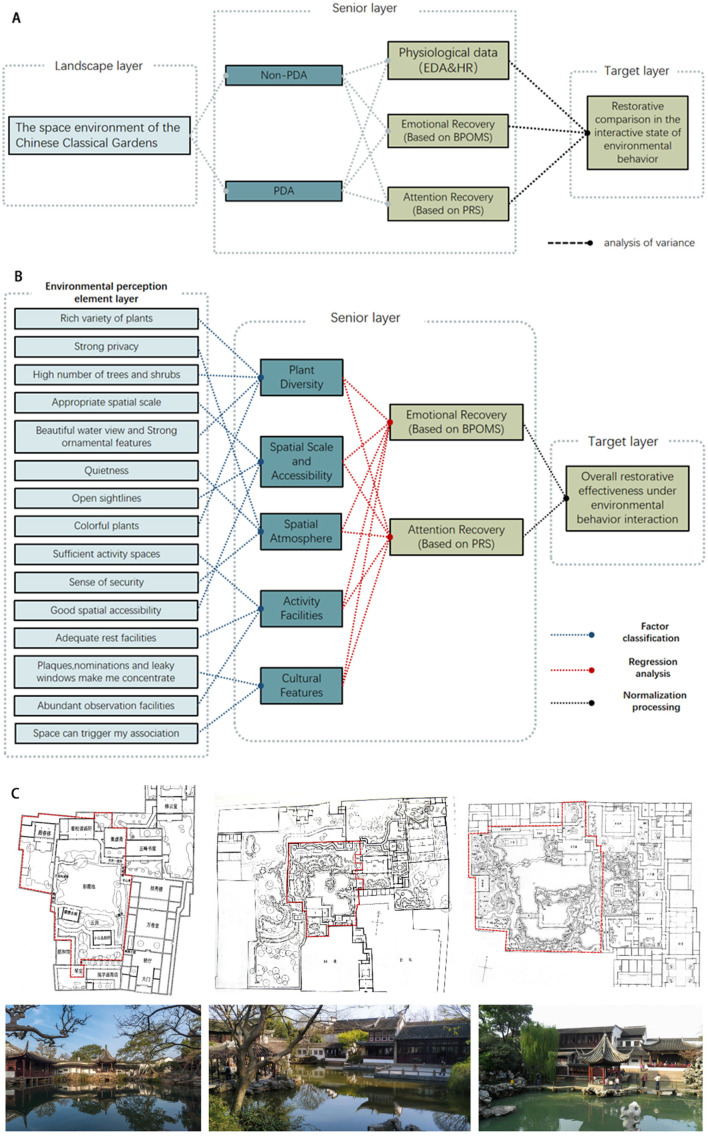
Two phases of the research and experiment areas. **(A)** Phase 1 research framework. **(B)** Phase 2 research framework. **(C)** Experiment areas of the Master of Nets Garden, Lingering Garden, and Lion Grove Garden.

The study aims to explore the impact the independent variable, “PDA in classical gardens”, imposes on restorative effects. To facilitate future environment design, we also try to identify the spatial features of the restorative environment such activities favor. Hence, this is a two-part study. Phase 1 (as Figure 1A shows): Quantify and compare restorative effects by collecting physiological and psychological data; Phase 2 (as Figure 1B shows) focuses on the perception of restorative environmental features that influence PDA; through statistical methods, it summarizes the dimensions and their feature factors influencing the formation of PDA, based on which the formation mechanism of PDA and PDA's relationship with restorative effects are explored.

### 2.1 Case studies

Located along the southern Yangtze River, three classical gardens that have made the World Heritage List, including the Master of Nets Garden, the Lingering Garden and the Lion Grove, were chosen for examination. Typical of their kind, moderately scaled, and stylistically distinctive, these gardens make good objects of study. Our research focused on college students, as they have long been an overly stressed group in need of mental care, making them an ideal demographic for our study. Therefore, a total of 90 students, aged between 18 and 25, were voluntarily and randomly recruited from a university. Among them, 24 were male and 66 female; 50 were design major and 40 were not. All participants were physically and mentally healthy. For more accurate results, testees were only allowed to move about within the pre-set water-centered areas (as Figure 1C shows).

### 2.2 Indicators and questionnaire

#### 2.2.1 Indicators

##### 2.2.1.1 Physiological indicators

Physiological indicators involved in this experiment include heart rate (HR) and electrical dermal activity (EDA). According to stress recovery theories, when stress is lessened, physical health will be restored, resulting in a decreased heart rate (16, 17); emotional arousal can be measured physiologically using EDA, which shows itself as the electrical changes on the skin's surface as the skin receives innervation signals from the brain (18, 19). EDA collects data that change both slowly and rapidly, called the basal activity-skin conductance level (SCL) and the phase activity-skin conductance response (SCR), respectively. The SCR data indicates a superior emotional recovery effect and is frequently employed as an indicator of emotional arousal (20). A wearable medical device, Empatica4 (E4), was used to collect those physiological data in real-time. In 2019, researchers from the University of Salzburg, Zurich University Hospital, Harvard University, Groningen University, and the University of Birmingham conducted a joint experiment using E4 (25). Their study tested stress variations in participants across different scenarios, which validated E4′s effectiveness as a reliable tool for physiological monitoring.

##### 2.2.1.2 Psychological indicators

Two popular psychological indicators, the BPOMS (Brief Profile of Mood States) and the PRS (Perceived Environmental Restoration Scale), are used to evaluate the psychological benefits of the gardens. The former measures a series of dimensions of mood swings; higher scores indicate healthier emotional states (21). The latter measures the four components of perceived environmental restorativeness and thus indicates how the environment affects attentional recovery (22).

#### 2.2.2 Questionnaire design

Our questionnaire consists of three sections. The first includes sections on personal information and choices of PDA, such as gender, age, literacy, self-evaluation of health state, and PDA design. Participants' activities are classified into nine categories, such as solitude (getting away from people), intimate communication, meditation, and book reading.

The second section focuses on the evaluation of the restorativeness of non-PDA, using the Brief Mood States Scale and the Subjective Restorativeness Scale, which are to measure emotional and attention restoration after non-PDA.

The third section features the evaluation of the restorativeness of PDA and the identification of spatial environmental characteristics. It includes the Brief Mood States Scale, the Subjective Restorativeness Scale, and the Spatial Environmental Characteristics Evaluation Questionnaire. This part mainly collects data on emotional and attention restoration after engaging in PDA and assesses subjects' perception of the spatial environment during the activities. The spatial environmental characteristics evaluation questionnaire adopts the Likert 7-point scale.

### 2.3 Experiment design

To test for restorative changes in behavioral activities in real-life circumstances, we designed a natural experiment to collect psychological and physiological data. The experiment was conducted multiple times on the mornings and afternoons from May to August 2021. To minimize the potential impact of such variables as temperature, humidity, environmental noise, and crowd density on the participants' psychological states and the quality of their experiences, the experiments were conducted only on comfortable, sunny days with similar temperatures. Additionally, weekends were avoided to minimize the impact of high foot traffic in the gardens. Each experiment took one experimentee and 43 min to finish, including 3 min for collecting baseline physiological data, 15 min for each of the two activities, and 10 min for questionnaire answering. The experiment can be divided into three phases:

1) Pre-experiment Phase: The experimentee was led to the vicinity of the experiment area and instructed to relax to restore their best psychological and physiological conditions. Then, the experimentee was informed in detail of the procedure while filling out the first section of the questionnaire, but the purpose of the experiment remained unveiled. Having reached an adequately calm state, the experimentee was tested for 3 min wearing an E4 bracelet to measure two physiological indicators, and the mean values were used as baseline values for the upcoming analyses.
2) Experiment Phase: The experimentee entered the experiment area of the garden for a 15-min of regular non-PDA, preferably nature-embracing, leisure activity, familiarizing themselves with the environment. Then, having completed the second part of the questionnaire, the experimentee selected a location to engage in a pre-selected PDA, during which time observation and recording were conducted from a distance to avoid interference. Throughout the experiment, a GPS app was utilized to record the experimentee's real-time spatial movement.
3) Post-experiment Phase: Having completed the activities, the experimentee removed the E4 bracelet and completed the last section of the questionnaire, rating their experience.

### 2.4 Data processing and analyses

**Figure 2:**
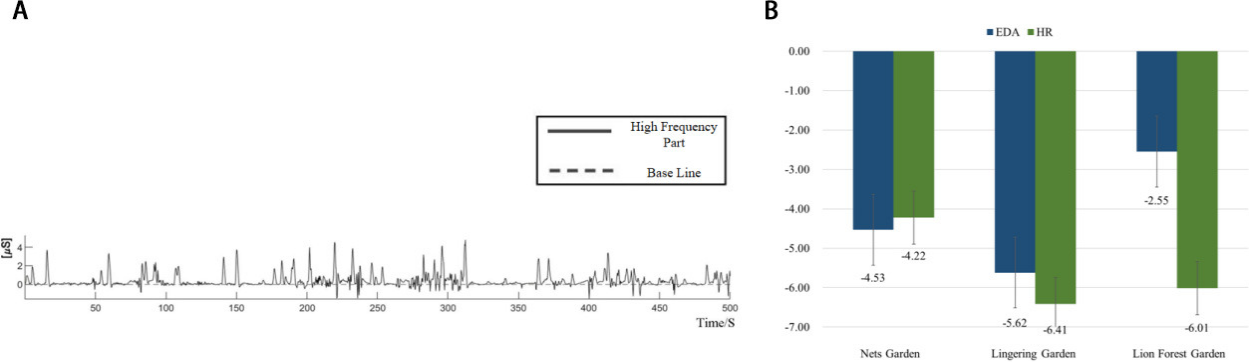
Physiological signal processing and results. **(A)** High-frequency EDA signal graph (baseline components removed). **(B)** Changes in physiological indicators before and after PDA.

Electra Dermal Activity (EDA) data were processed by using Matlab, their curves enhanced by using LedaLab (as Figure 2A shows). Heart rate (HR) and psychological scale data were processed by EXCEL. These pre-processed physiological data, together with the psychological information sourced from the questionnaire, were then analyzed by using SPSS 25.0.

For better accuracy, the mean values of EDA and HR taken prior to the experiment were used as baseline values, and recovery was evaluated by comparing experiment values with baseline values. Factor analysis was employed to classify questionnaire data on spatial environmental features. Paired *t*-tests were utilized to compare the differences in mean values of various indicators before and after PDA. One-way analysis of variance (ANOVA) was used to compare the differences in the changes of various indicators among the three classical gardens. Multiple regression and normalization analysis were employed to explore the contribution of spatial environmental features to the restorative effects.

## 3 Identification of restorative environmental factors and evaluation of restorative effects

Because the one-way ANOVA results (P1 = 0.826 > 0.05, P2 = 0.915 > 0.05) reveal no significance of the EDA and HR data (baseline values) collected prior to the experiment, the restorative effects observed during later stages of the experiment can be seen as a result of visitor activity in the garden area. According to the Perceptual Resilience Scale (PRS) data, both activities yielded scores higher than 3.5 (the scale's midpoint), meaning leisure activities in a traditional garden generate significant restorative results. The restorative effects of the two types of activities will be revealed below.

### 3.1 Evaluation of restorative effects

We compared data collected before and after the experiment to determine the level of physical and mental recovery activities in the gardens may generate, using the one-way ANOVA analysis.

#### 3.1.1 Physiological indicators

Leaving out six obviously faulty entries, we conducted the *t*-test on the rest 84 sets of physiological data. As the results reveal (as Table 1 shows), the subjects' EDA and HR in the three classical gardens dropped after the PDA, and such a decline was statistically significant (*p* < 0.05). The Lingering Garden generated the greatest decrease (5.62) in EDA, followed by the Mater of Nets Garden, and then by the Lion Grove (as Figure 2B shows); the Lingering Garden generated the greatest decrease (6.41) in HR, followed by the Lion Grove, and then by the Master of Nets Garden.

**Table 1:** *T*-test and *P*-value of physiological indicators before and after PDA.

**Physiological indicators**	**Experimental scenes**	**Pre-measured values**	**Post-measured values**	***P*-value (95% CI)**
EDA	Nets garden	1.10 ± 5.36	−3.43 ± 8.03	0.000
	Lingering garden	9.08 ± 7.76	3.46 ± 5.12	0.000
	Lion forest garden	5.17 ± 5.87	2.62 ± 4.33	0.002
HR	Nets garden	2.71 ± 11.72	−1.51 ± 10.59	0.008
	Lingering garden	10.35 ± 12.24	3.94 ± 11.84	0.000
	Lion forest garden	12.01 ± 9.76	6.00 ± 11.18	0.015

Please note that some subjects unexpectedly reported increased EDA and HR values after the experiment, indicating a state of arousal during activities. However, unlike negative arousal such as anger, stress or fear, such arousal was positive, resulting from the high levels of pleasure one experienced appreciating the culturally and naturally enjoyable elements of the gardens. This suggests that EDA and HR data alone do not tell the full story about restorative effects, meaning we need to also incorporate mental indicators for enhanced analyses.

#### 3.1.2 Mental indicators

According to our findings (as Table 2 shows), in all three gardens, Degree of Distance, Fascination, and Coherence all exhibit an increase after the PDA, while a decrease in Harmony is observed; Degree of Distance increased the most (by 0.60) in the Lingering Garden; Coherence increased the most (by 0.52) in the Master of Nets Garden and Lingering Garden; Fascination considerably increased (by 0.16) in the Master of Nets Garden, representing a significant difference (*P* = 0.03); and Harmony decreased the most (by 0.40) in the Lingering Garden, although not representing a significant difference.

**Table 2:** *T*-test and *P*-value of psychological indicators before and after PDA.

**Psychological indicators**	**Experimental scenes**	**Pre-measured values**	**Post-measured values**	***P*-value (95% CI)**
Degree of distance	Nets garden	5.567 ± 0.74	6.03 ± 0.76	0.000
	Lingering garden	5.33 ± 0.77	5.93 ± 0.94	0.002
	Lion forest garden	5.58 ± 0.88	6.17 ± 0.80	0.000
Charisma degree	Nets garden	4.28 ± 0.41	4.44 ± 0.33	0.030
Consistency	Nets garden	4.55 ± 0.36	4.774 ± 0.36	0.000
	Lingering garden	5.33 ± 0.69	5.85 ± 0.75	0.000
	Lion forest Garden	5.49 ± 0.70	5.89 ± 0.74	0.000
Tension value	Nets garden	1.76 ± 0.37	1.32 ± 0.30	0.000
	Lingering garden	1.64 ± 0.31	1.40 ± 0.30	0.001
	Lion forest garden	1.960 ± 0.43	1.462 ± 0.33	0.000
Anger value	Nets garden	1.43 ± 0.28	1.15 ± 0.19	0.000
	Lingering garden	1.35 ± 0.26	1.24 ± 0.26	0.020
	Lion forest garden	1.45 ± 0.36	1.22 ± 0.19	0.001
Fatigue value	Nets garden	1.85 ± 0.56	1.47 ± 0.48	0.000
Depression value	Nets garden	1.62 ± 0.36	1.24 ± 0.24	0.000
	Lingering garden	1.49 ± 0.37	1.28 ± 0.25	0.004
	Lion forest garden	1.58 ± 0.35	1.39 ± 0.26	0.003
Panic value	Nets garden	3.56 ± 0.59	3.78 ± 0.54	0.025
	Lingering garden	1.86 ± 0.46	1.58 ± 0.41	0.002
	Lion forest garden	2.02 ± 0.44	1.7 ± 0.38	0.001
Energetic value	Nets garden	1.83 ± 0.44	1.45 ± 0.33	0.000
Self-esteem value	Nets garden	3.04 ± 0.60	3.29 ± 0.49	0.042
	Lingering garden	3.13 ± 0.36	3.32 ± 0.48	0.015

**Figure 3:**
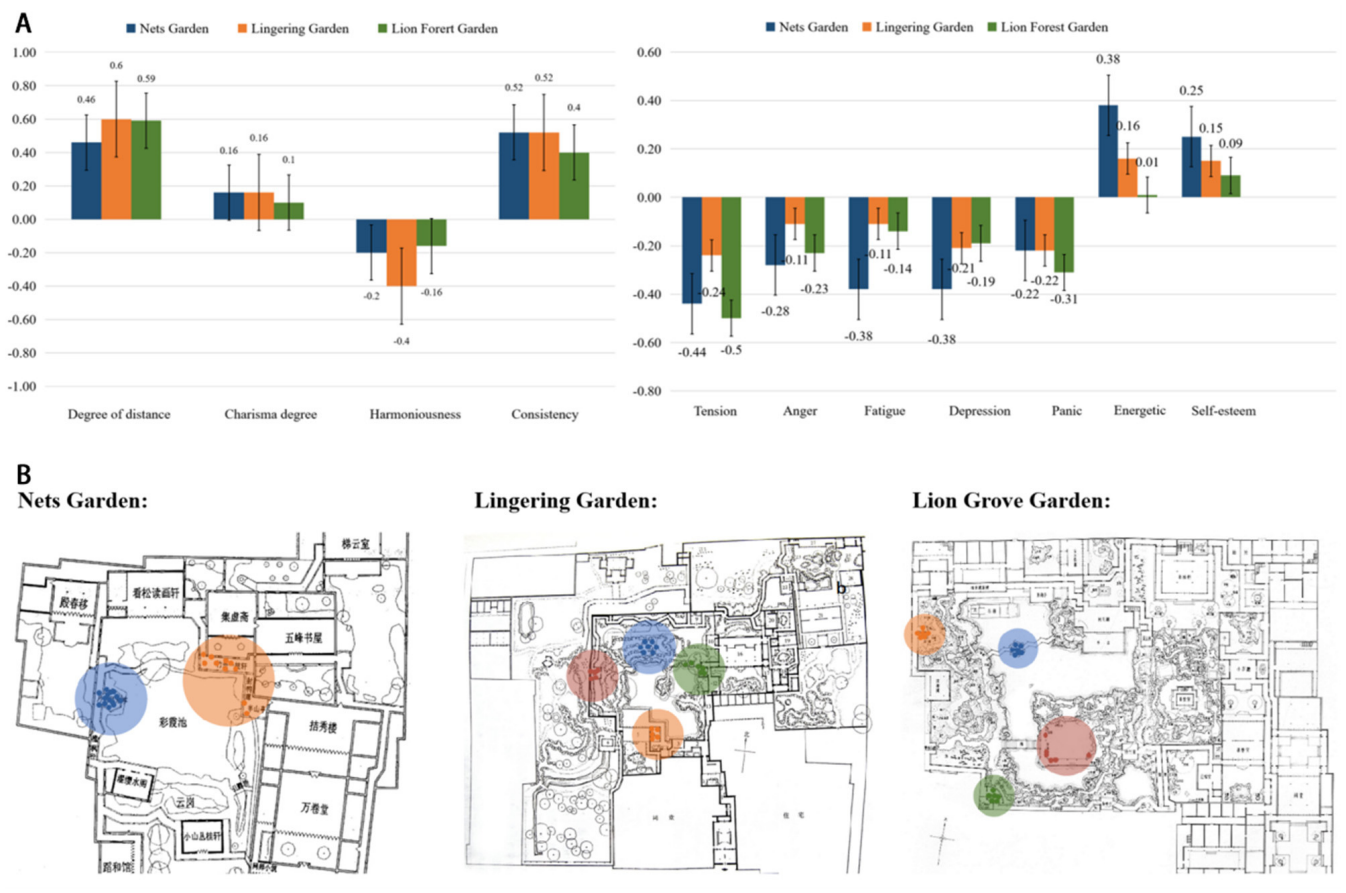
Comparison of psychological evaluations and analysis of PDA preferences. **(A)** Changes in PRS and BPOMS scale before and after PDA. **(B)** The distribution of PDA preferences in three gardens.

According to the results from a paired *t*-test on BPOMS data (as Table 2 shows), after the PDA, all three gardens witnessed a fall in negative emotions and a rise in PDA. With negative emotions, all three gardens showed a significant drop (*P* < 0.05) in tension, anger, depression, and panic, the Lion Grove generating the greatest, very significant (*P* < 0.01) decrease in tension and panic values, at 0.5 and 0.31, respectively (as Figure 3A shows). As for positive emotions, Energy values increased highly significantly (*P* = 0.000) by 0.38 in the Master of Nets Garden, and Self-esteem increased significantly (*P* < 0.05) in the Master of Nets Garden and the Lingering Garden, the Master of Nets Garden marking the largest increase (0.25).

The statistical results indicate that, when it comes to mental restorativeness, both PDA and non-PDA can promote attention restoration, alleviate negative emotions, and enhance positive emotions. Although the increase in positive emotions in the Lion Grove sample is not statistically significant, overall, the psychological restorative effects of PDA still appear to be greater than those of non-PDA.

### 3.2 PDA location preference

As is discovered, there were two preferred locations for PDA in the Master of Nets Garden, four in the Lingering Garden, and four in the Lion Grove Garden. The two “hotspots” of the Master of Nets Garden include the Yuedao Fenglai Pavilion (favored by 16 out of 30 subjects) and the Zhuwai Yizhi Pavilion and Sheya Gallery area (choice of 10 out of 30). The four favored areas of the Lingering Garden include Wenmuxiangxuan (5 out of 30), Keting (7 out of 30), Qingfengchi Hall (6 out of 30), and Mingse Tower (5 out of 30). The four favored areas of the Lion Grove Garden include the Tingtao Pavilion (6 out of 30), the Huxin Pavilion (7 out of 30), the Wisteria Stand (6 out of 30), and the Fan Pavilion (6 out of 30) (as Figure 3B shows).

### 3.3 Analysis of environmental characteristics perception factors affecting the occurrence of PDA

The influence of different spatial environmental perception on the occurrence of PDA was examined. By using SPSS, the KMO value was found to be 0.685, and the Sig. value of Bartlett's sphericity test was 0.000, verifying the data for factor analysis. We then identified five major factors (“dimensions”) whose eigenvalues were >1. A cumulative contribution rate of 66.851% for the principal components was observed, indicating that these five factors were decisive. To group all factors into the five categories, the data was rotated using the maximum variance rotation method to produce factor loading coefficient values, based on which we put each factor into a suitable category—when the absolute value of the factor loading coefficient is >0.4, it indicates a corresponding relationship between the category and the factor (23, 24). As Table 3 shows, the spatial environmental features can be classified into five dimensions: (1) Plant Diversity, which includes four factors; (2) Scale and Accessibility, which includes three factors; (3) Space Atmosphere, which includes three factors; (4) Event Facilities, which includes three factors; (5) Cultural Features, which includes two factors.

**Table 3:** Factor analysis of spatial environment characteristics questionnaire.

**Factor category**	**Evaluation item**	**Loading factor**				
		**1**	**2**	**3**	**4**	**5**
Plant diversity (1)	Rich variety of plants	**0.871**	−0.02	0.045	−0.036	0.097
	Colorful plants	**0.798**	−0.012	0.013	0.099	−0.048
	High number of trees and shrubs	**0.752**	0.088	0.027	0.116	0.117
	Beautiful water view and Strong ornamental features attractive	**0.485**	0.431	−0.131	0.213	0.025
Spatial scale and accessibility (2)	Appropriate spatial scale	0.049	**0.789**	0.122	0.171	0.092
	Open sightlines	0.110	**0.787**	−0.184	−0.053	0.174
	Good spatial accessibility	−0.074	**0.649**	0.333	0.051	0.013
Spatial atmosphere (3)	Strong privacy	0.140	−0.012	**0.804**	0.022	−0.003
	Quietness	0.048	0.289	**0.796**	−0.006	0.003
	Sense of security	−0.042	−0.093	**0.714**	0.178	0.275
Activity facilities (4)	Sufficient activity spaces	−0.030	0.009	−0.073	**0.824**	0.180
	Adequate rest facilities	0.180	0.220	0.097	**0.778**	−0.169
	Abundant observation facilities	0.170	0.017	0.217	**0.752**	0.274
Cultural features (5)	Space can trigger my association	0.101	0.142	0.017	−0.006	**0.837**
	Plaques nominations and leaky windows make me concentrate	0.051	0.101	0.179	0.243	**0.718**

Bold indicates a statistically significant relationship between the item and the category to which it belongs.

## 4 Analyses of privacy preferences and restorative effects based on environmental feature factors

### 4.1 Evaluation of restorative environmental features for high-frequency locations

**Figure 4:**
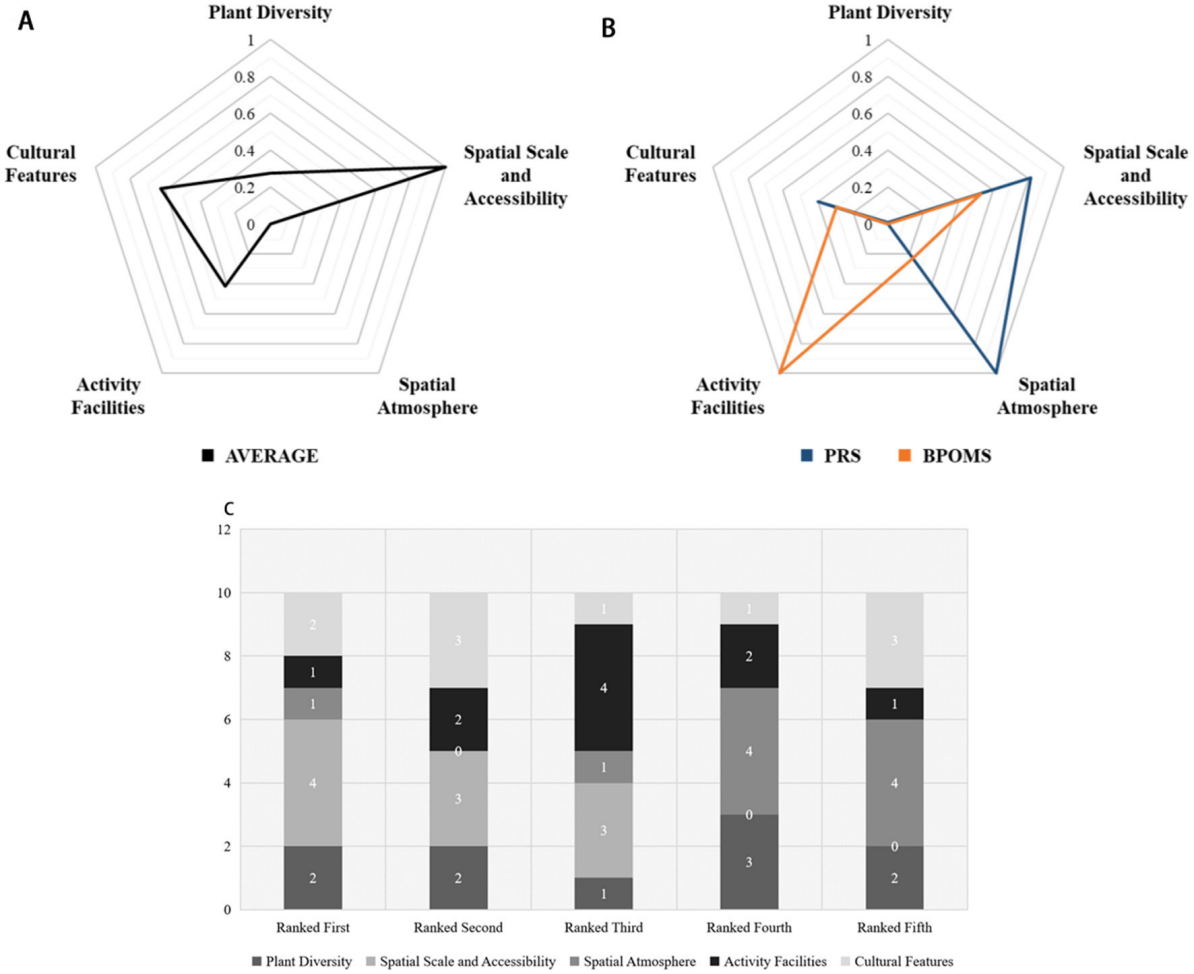
Analyses of privacy preferences and restorative effects based on environmental feature factors. **(A)** Environmental perception evaluation score mean normalization processing. **(B)** Standardization coefficient absolute value normalization. **(C)** The times of environmental perception factor dimensions of 10 high-frequency stagnation points in each ranking.

We assess the environmental features using the Likert 7-point scale method. Based on the questionnaire results, we calculate the average scores for the perceived environmental features of the ten high-frequency locations. These averages are normalized and presented in a radar chart (as Figure 4A shows). The findings reveal variations in scores across environmental perception factors in high-frequency locations, indicating that different environmental features exert varying degrees of influence on individuals when people select locations for their PDA. Subsequently, we rank and compare the environmental perception factors of the ten high-frequency locations, compiling a stacked bar chart (as Figure 4C shows) to depict the quantity of environmental perception factors in different ranking positions. When we compare it to Figure 4A, it becomes evident that the dimensions of “Spatial Scale and Accessibility” receive the highest evaluation scores and hold the most top-ranking positions. This fact suggests that the settings featuring “appropriate spatial scale,” “open sightlines,” and “good spatial accessibility” are most likely to attract participants for highly restorative activities.

### 4.2 Multivariate regression analysis of restorative environmental perception scores and restorative effects for high-frequency locations

To study the influence of different spatial environmental characteristics on the preference for locations of privacy-related activities, we first conducted a KMO and Bartlett's test of sphericity using SPSS. The results showed a KMO value of 0.685 and a Bartlett's test significance (Sig.) value of 0.000, indicating that the data was suitable for factor analysis. Using an eigenvalue >1 as the extraction criterion, we identified five factors, with a cumulative variance contribution of 66.851%, demonstrating a significant effect. This indicates that these five factor dimensions are meaningful. To facilitate the identification of the relationships between factors and research items, we employed the maximum variance rotation method. Based on the factor loading coefficient values obtained, we further identified the correspondence between the five factor dimensions and the specific sub-factor items. The results, displayed in Figure 4B, illustrate that different environmental dimensions exert varying degrees of influence on individual restorative effects, encompassing cognitive and emotional recovery. Notably, the “Spatial Atmosphere” dimension (characterized by “strong privacy,” “quietness,” and “sense of security”) exerts the most significant influence on cognitive recovery, while the “Activity Facilities” dimension (including “sufficient activity spaces,” “adequate rest facilities,” and “abundant observation facilities”) exerts the most substantial impact on emotional recovery.

### 4.3 Analysis results

To sum up, it is highlighted that the subjective evaluation scores for various dimensions of environmental characteristics in classical gardens may not entirely reflect the weight of their impact on restorative effects. It suggests that individuals' cognitive behavioral preferences change between the stages of private formation and PDA. When searching for private points, people tend to use visual cognition of the environment, favoring locations with suitable spatial scale and unique cultural characteristics. In contrast, during PDA, individuals focus more on the cognitive aspects of the space, emphasizing its atmosphere, privacy, and the quantity and arrangement of activity facilities. This phenomenon is explained by combining insights from environmental behavior studies and environmental psychology.

## 5 Conclusions

### 5.1 Positive restorative effects of PDA in classical gardens

The study shows that diverse activities touring the spatial settings of classical gardens can effectively promote restorativeness. The restorative effects stimulated by environmental experiences are the results of both environmental perception and behavioral experiences. By comparing the key restorative indicators between PDA and non-PDA, the study further discovers that, compared to non-PDA, PDA demonstrate better restorative performance in terms of physiological, psychological, and restorative scale statistical data. It should be noted that, while the overall statistical analyses support the above conclusions, the data from the Mater of Nets Garden case does not show significant results in terms of positive emotional recovery. This may be due to the garden's characteristic feature of abundant rockeries with insufficient greenery; a larger sample size may help clarify the hypothesis. We plan to further investigate this phenomenon.

### 5.2 Environmental feature factors affecting PDA in classical gardens

This article conducts trajectory sampling and spatial perception factor extraction for PDA within three classical gardens. It performs factor analysis on the perceived environmental features, resulting in the identification of five key environmental factor dimensions influencing the formation of PDA. Among them, the “Spatial Scale and Accessibility” dimension has the greatest impact on the choice of “Preferences for PDA” and serves as the primary inducement for the formation of private spots. On the other hand, the environmental factor dimensions with the greatest impact on the “Restorative Effects” are “Spatial Atmosphere” and “Activity Facilities”. It is also discovered that PDA and the restorative effects lead to significantly different impact weight rankings of environmental factor dimensions. This means that further research is needed to explain the mediating role of behavior in the relationship between environmental stimuli and restorative effects, as well as to explore the underlying mechanisms.

### 5.3 Discussion

As mentioned earlier, the weight rankings of environmental factor dimensions in the pathways of “Preferences for PDA” and “Restorative Effects” exhibit significant differences among the three case gardens. This indicates that behavioral activities mediate the relationship between environmental factor dimensions and restorative effects, which aligns with many previous studies. In the future, we will further consider behavioral activities as a mediating variable to systematically investigate the comprehensive impact pathways and mechanisms of environmental characteristics, behavioral mediation on restorative effects. This will contribute to the development of more refined and science-based approaches to restorative environment design. Notably, this study has several limitations. For instance, the sample population consists entirely of college students. While they are representative of the mentally troubled groups in some respects, the homogeneity of the group differs significantly from the diversity of the real world, which may compromise the validity of the conclusions to some extent. Also, the experimental subjects, experimental precision, and a limited number of experimental samples in this study could have had an impact on the results, because statistical methods such as factor analysis, correlation analysis, and regression analysis are sensitive to sample size. Many environmental factor dimensions with lower effect sizes, as well as factors influencing restorative effects, may have been excluded from this study. The content and structure of the section exploring the factors require further enhancement. Therefore, in the future, it is necessary to expand the sample size and systematically consider various environmental factors while incorporating demographic factors such as age, educational level, and gender into the restorative effects model.

## Data Availability

The original contributions presented in the study are included in the article/supplementary material, further inquiries can be directed to the corresponding authors.
